# Educational Workplace Dietary Interventions and Occupational Well-Being Among Healthcare Professionals: A Scoping Review

**DOI:** 10.3390/healthcare14172699

**Published:** 2026-08-24

**Authors:** Maria Antoniadou, Theodora Kalogerakou, Polyxeni Mangoulia, Theodoros Varzakas

**Affiliations:** 1 Department of Dentistry, School of Health Sciences, National and Kapodistrian University of Athens, 11527 Athens, Greece; tkalogerakou@hotmail.com; 2 Certified Systemic Analyst Program, University of Piraeus, 18534 Piraeus, Greece; 3 Department of Nursing, School of Health Sciences, National and Kapodistrian University of Athens, 11527 Athens, Greece; pmango@nurs.uoa.gr; 4 Department of Food Science and Technology, University of the Peloponnese, 24100 Kalamata, Greece; t.varzakas@uop.gr

**Keywords:** healthcare personnel, dentists, nutrition education, food habits, burnout, professional, occupational health, workplace health promotion, workplace nutritional resilience

## Abstract

**Background:** Educational workplace dietary interventions have emerged as a promising approach to supporting occupational well-being among healthcare professionals. This scoping review mapped and synthesized the available evidence on such interventions across healthcare settings. **Methods:** A scoping review was conducted in accordance with the PRISMA-ScR (Preferred Reporting Items for Systematic Reviews and Meta-Analyses Extension for Scoping Reviews) guidelines. PubMed, Scopus, and the Cochrane Library were searched for studies published between December 2020 and March 2026. Thirteen studies met the predefined eligibility criteria. **Results:** Across the included studies, healthcare professionals exhibited irregular eating patterns, meal skipping, and suboptimal dietary behaviours associated with occupational stress. Educational workplace interventions combining nutrition education with behavioural, organizational, and lifestyle components were associated with improved nutrition knowledge, dietary behaviours, selected health indicators, and occupational well-being. Thematic synthesis identified workplace organization, behavioural determinants, and organizational support as key influences on dietary practices and professional well-being. **Conclusions:** Workplace nutrition should be considered an integral component of occupational health rather than solely an individual lifestyle behaviour. The proposed Workplace Nutritional Resilience framework highlights the potential contribution of educational, behavioural, and organizational strategies to occupational well-being. Further longitudinal and intervention studies in specific healthcare professions are warranted, particularly among dental professionals.

## 1. Introduction

Healthcare professionals are frequently exposed to occupational stress, demanding working conditions, and irregular work schedules that may adversely affect dietary behaviours and occupational well-being. Among healthcare professions, dentistry represents a particularly demanding clinical environment [1,2,3,4]. Similar patterns of occupational stress and health-related challenges have also been documented across other healthcare professions, indicating that these concerns represent a broader issue within healthcare systems rather than an isolated problem affecting dentists alone [1,2,3,4,5]. In the post-pandemic period, increasing attention has been directed toward workplace interventions targeting dietary habits, stress management, and occupational well-being among healthcare professionals [6,7,8]. Extended working hours, demanding clinical schedules, and irregular routines have been associated with burnout, unhealthy dietary behaviors, and impaired occupational well-being among dentists [6].

In response to these occupational challenges, workplace dietary interventions have emerged as practical strategies to support both physical and psychological health among healthcare professionals. Previous evidence suggests that occupational stress is associated with unhealthy eating behaviors, emotional eating, and burnout, while nutrition-focused educational programmes may promote healthier dietary practices, improve food literacy, and support long-term psychological well-being [3,7,8,9,10,11,12,13]. Because dentists routinely work under sustained cognitive, emotional, and ergonomic demands, these interventions may be particularly relevant to dental practice.

Recent studies have reported substantial disruptions in eating behaviours among healthcare professionals, often associated with occupational stress, demanding work routines, and limited access to healthy food options [13,14]. Dentists and other healthcare professionals have been reported to exhibit emotional eating, irregular meal patterns, and increased consumption of energy-dense foods, frequently accompanied by anxiety, psychological distress, and occupational dissatisfaction [15,16,17,18]. Emerging evidence further suggests that workplace interventions integrating nutrition education, behavioral support, and stress-management strategies may contribute positively to healthier dietary behaviors, improved psychological well-being, and better stress-management outcomes among healthcare professionals [19,20,21,22,23,24].

To enhance their effectiveness, these interventions have increasingly been designed through interdisciplinary collaboration involving nutritionists, occupational health specialists, and dental educators, allowing adaptation to factors such as age, gender, workload, and occupational stress levels [1,18,25,26,27,28]. Particular attention has also been directed toward healthcare professionals exposed to irregular working schedules and prolonged working hours, as these occupational conditions have been associated with irregular meal timing, circadian disruption, emotional eating, and unhealthy dietary patterns [29,30,31]. Accordingly, workplace programs incorporating meal-planning guidance, improved access to healthy foods, nutrition education, and stress-management support have shown promising-although still limited-evidence for promoting healthier lifestyle behaviors and occupational well-being [6,32,33,34,35,36]. Emerging evidence increasingly supports the view that workplace nutrition should be regarded not merely as an individual lifestyle choice but as an integral component of occupational health and professional well-being within healthcare settings.

Recent studies have also emphasized the importance of integrating nutrition education and workplace wellness strategies within the broader professional development of healthcare workers [37,38,39]. Despite growing interest in workplace nutrition and occupational well-being, synthesized evidence remains fragmented, while evidence specific to dental professionals is particularly limited [40,41,42]. Although many of the available studies have been conducted among broader healthcare populations, dentistry represents a distinctive professional environment characterized by prolonged static postures, tightly scheduled clinical appointments, continuous patient interaction, high cognitive demands, limited opportunities for regular breaks, and substantial ergonomic and emotional stress. These profession-specific characteristics may influence dietary behaviors, eating patterns, and occupational well-being differently from other healthcare settings. Therefore, synthesizing the available evidence from both dental and broader healthcare populations provides an important foundation for understanding workplace nutrition within dentistry while simultaneously highlighting the need for future dentist-specific research. To address this evidence gap, the present scoping review maps and synthesizes the available evidence on educational workplace dietary interventions among healthcare professionals while identifying evidence gaps in specific professional groups, particularly dental professionals.

The objectives are to identify commonly applied intervention approaches, examine associations between workplace nutrition strategies and occupational well-being outcomes, and propose future directions for research and workplace health promotion among healthcare professionals. Specifically, the review addresses the following research question: “Which educational workplace dietary interventions are associated with healthier dietary behaviours, stress management, and occupational well-being among healthcare professionals?”

## 2. Materials and Methods

### 2.1. Study Design

This scoping review was conducted in accordance with the Preferred Reporting Items for Systematic Reviews and Meta-Analyses Extension for Scoping Reviews (PRISMA-ScR) guidelines. A scoping review methodology was considered appropriate because the available evidence was heterogeneous in terms of populations, interventions, and outcomes, making an evidence-mapping approach more suitable than an effectiveness-focused review. Accordingly, the review examined educational workplace dietary interventions and their potential associations with occupational well-being among healthcare professionals, while identifying evidence gaps relevant to specific professional groups, particularly dental professionals. A comprehensive electronic literature search was performed in PubMed, Scopus, and the Cochrane Library to identify relevant studies published between December 2020 and March 2026. This publication period was selected to capture contemporary evidence on workplace nutrition and occupational well-being during a time of increased scientific interest in these topics. The review was not designed to evaluate the direct effects of the COVID-19 pandemic; therefore, COVID-19-related search terms were not incorporated into the search strategy, as the review question focused on educational workplace dietary interventions and their associations with occupational well-being rather than on pandemic-specific outcomes. No review protocol was registered or published for this scoping review.

### 2.2. Search Strategy

The search strategy combined Medical Subject Headings (MeSH) and free-text terms related to healthcare professionals, including dentists, workplace nutrition, dietary behaviors, occupational stress, burnout, resilience, well-being, and educational interventions. Boolean operators (AND/OR) were used to optimize retrieval of relevant studies. The complete search strategies for all databases are presented in the Supplementary Material. The PubMed search strategy was structured as follows: (dentist OR dental professional OR dental staff OR oral healthcare worker OR nurses OR healthcare OR doctors) AND (nutrition OR diet OR dietary behavior OR eating habits OR food literacy OR nutrition education OR workplace nutrition) AND (burnout OR stress OR resilience OR mental health OR occupational health OR wellbeing OR fatigue) AND (intervention OR program OR education OR training OR counselling OR behavior change OR strategy).

### 2.3. Eligibility Criteria

Eligibility criteria were developed according to the Population–Concept–Context (PCC) framework recommended for scoping reviews.

- Population: Healthcare professionals, including dentists and dental professionals, working in clinical, academic, educational, or healthcare-related settings.
- Concept: Educational workplace dietary interventions, nutrition education programmes, food literacy initiatives, workplace wellness programmes, structured meal-planning strategies, and other workplace-based approaches aimed at improving dietary behaviours, nutrition-related knowledge, and occupational well-being.
- Context: Workplace, occupational, educational, or healthcare environments relevant to healthcare professionals.

Studies involving diverse healthcare populations were included when they addressed dietary behaviours, nutrition-related outcomes, occupational stress, burnout, resilience, psychological well-being, or workplace health outcomes relevant to healthcare professionals. Where appropriate, the findings were also considered for their potential relevance to dental practice. Eligible study designs included randomized controlled trials, quasi-experimental studies, cohort studies, case–control studies, cross-sectional studies, pilot interventions, educational studies, qualitative studies, mixed-methods studies, and observational research. Only articles published in peer-reviewed journals and written in English were considered. Reviews, editorials, commentaries, conference abstracts, protocols, and publications lacking sufficient information regarding educational dietary interventions or occupational well-being outcomes were excluded.

### 2.4. Study Selection and Data Extraction

Two reviewers independently screened titles, abstracts, and full-text articles according to the predefined eligibility criteria. Discrepancies were resolved through discussion and consensus. Data extraction was performed using a standardized form and included information on authorship, year of publication, country, study design, population characteristics, intervention characteristics, educational components, outcome measures, and principal findings related to dietary behaviors, nutrition knowledge, stress, burnout, resilience, and occupational well-being.

### 2.5. Data Synthesis

Consistent with the objectives of a scoping review, the purpose of this study was to map the existing literature rather than evaluate intervention effectiveness or generate pooled effect estimates. Therefore, no formal risk-of-bias assessment or methodological quality appraisal was undertaken, as such assessment was not considered appropriate for the objectives of this review. Extracted data were synthesized descriptively and narratively. Studies were grouped according to study design, population characteristics, intervention type, educational strategies, and reported occupational well-being outcomes. Recurring concepts, intervention characteristics, and reported outcomes were compared across the included studies and iteratively organized into thematic categories that reflected common patterns identified during the synthesis. Because the included studies represented diverse healthcare populations and settings, the synthesis focused on identifying recurring patterns and transferable themes rather than profession-specific conclusions. The thematic synthesis followed an inductive approach, whereby themes emerged iteratively from the extracted data rather than being based on a predefined analytical framework. The thematic organization was developed collaboratively by the review team through discussion and consensus to ensure consistency in the interpretation of the identified patterns. Particular attention was given to identifying research trends, methodological characteristics, evidence gaps, and areas requiring further investigation. Consequently, the findings should be interpreted as a descriptive and thematic synthesis of the available evidence, intended to identify recurring patterns, emerging themes, transferable insights, and knowledge gaps rather than determine the strength, certainty, or effectiveness of the evidence. Accordingly, the methodological characteristics and reported study limitations were extracted for descriptive purposes only and should not be interpreted as a formal critical appraisal or risk-of-bias assessment.

## 3. Results

**Figure 1:**
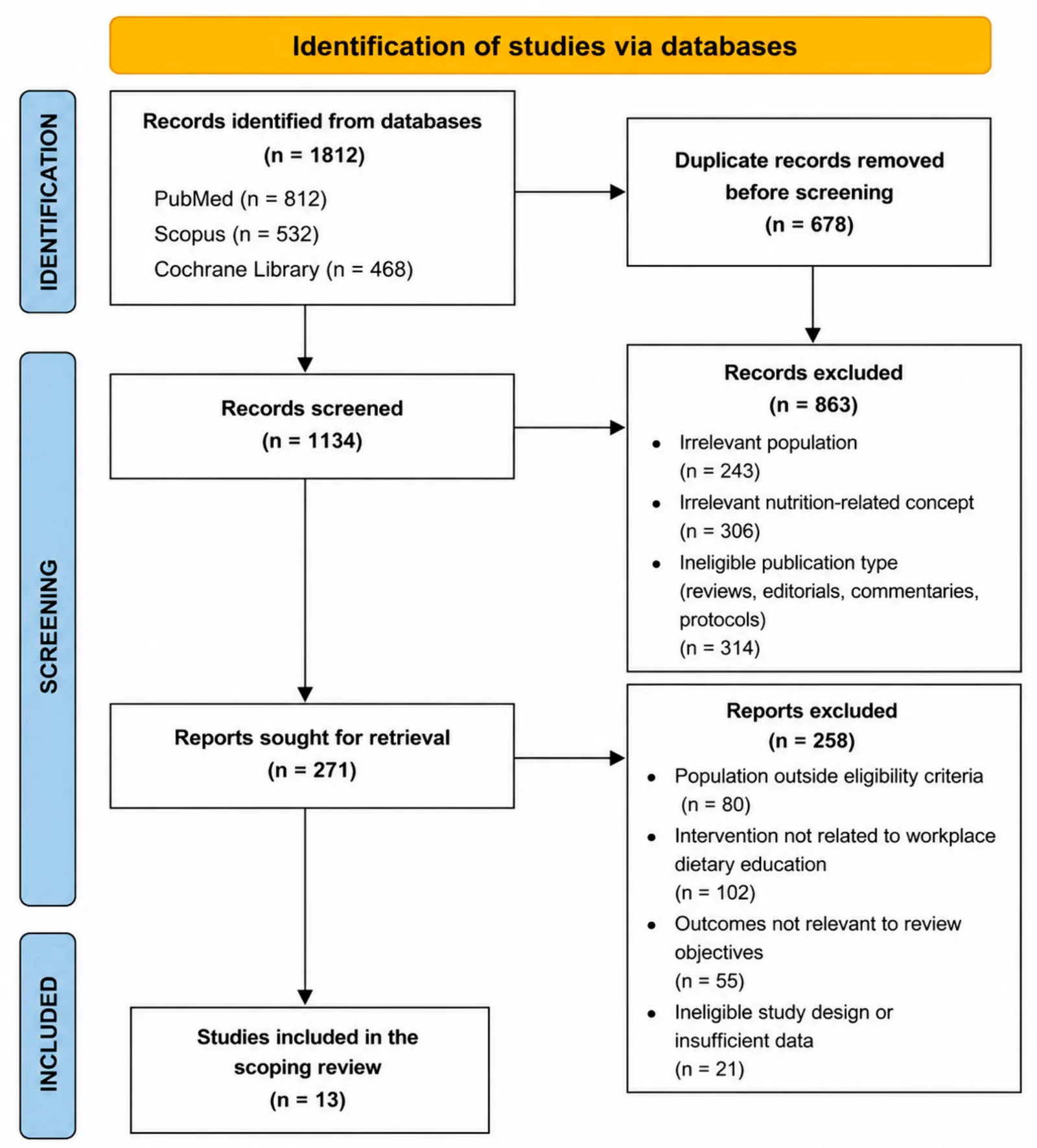
PRISMA-ScR flow diagram of the study selection process.

A total of 1812 records were identified through electronic database searching, including PubMed (n = 812), Scopus (n = 532), and the Cochrane Library (n = 468). After removal of 678 duplicate records, 1134 records remained for title and abstract screening. Following screening, 271 reports were sought for retrieval and assessed for eligibility. Of these, 258 reports were excluded because of irrelevant population (n = 80), intervention mismatch (n = 102), non-relevant outcomes (n = 55), or ineligible study design or publication type (n = 21). Ultimately, 13 studies met the eligibility criteria and were included in the final evidence synthesis. The study selection process is presented in Figure 1.

The completed PRISMA-ScR checklist is provided in the Supplementary Material (Table S1).

Table 1 presents the characteristics of the studies included in this scoping review.

Given the substantial methodological heterogeneity of the included studies, including observational, qualitative, educational, and intervention-based designs, a formal quantitative quality scoring approach was not considered appropriate for the objectives of this scoping review. Instead, methodological characteristics and study limitations were extracted and reported descriptively in Table 1 to facilitate interpretation of the evidence base.

### 3.1. Summary of Included Studies

A total of thirteen studies met the eligibility criteria and were included in the final synthesis. The evidence base comprised a heterogeneous mix of cross-sectional studies, qualitative investigations, educational interventions, workplace nutrition programs, and observational research conducted across healthcare and academic settings. For the purposes of evidence mapping, reported outcomes were considered within the following broad domains: nutrition knowledge, dietary behaviours, occupational stress and burnout, psychological and occupational well-being, and intervention-related outcomes. Despite the diversity of study designs and populations, the relatively small number of eligible studies highlights the limited evidence currently available in this field. Study populations included dentists, dental professionals, physicians, nurses, healthcare workers, and healthcare students from Europe, Asia, Africa, Australia, North America, and South America [14,15,24,26,35,38,43,44,45,46,47,48,49]. Among the included populations, only two studies focused specifically on dental professionals, representing different educational and clinical practice environments as reported by the original studies. Across the included studies, the primary areas of investigation were workplace dietary behaviours, nutrition-related knowledge and education, occupational stress, burnout, resilience, psychological well-being, and workplace nutrition interventions. Several studies examined the impact of the COVID-19 pandemic on dietary habits and occupational health, reporting associations between workplace stress, unhealthy eating behaviours, and reduced well-being [14,15,43], whereas others focused on nutrition literacy, food-related attitudes, educational interventions, and workplace nutrition programs designed to promote healthier dietary practices and support occupational health outcomes [26,38,46,49]. Overall, the included studies demonstrated considerable methodological heterogeneity with respect to study design, participant characteristics, intervention components, and outcome measures, limiting direct comparison across studies [14,24,35,43]. In addition, only two studies focused specifically on dental professionals, including dental students, practicing dentists, and members of the dental team, highlighting the limited profession-specific evidence available. These studies were conducted in mixed educational and clinical settings, including dental schools, public and private dental services, and specialist public hospital services [26,47]. Furthermore, longitudinal or controlled intervention studies remained scarce, with only a limited number of workplace intervention studies identified [46,49].

### 3.2. Type of Study and Study Design

The included studies comprised predominantly cross-sectional studies, supplemented by educational intervention studies, pilot pre–post intervention studies, qualitative studies, and one randomized controlled trial [14,15,24,26,35,38,43,44,45,46,47,48,49]. Cross-sectional studies represented the largest proportion of the evidence base, whereas intervention studies were comparatively limited and generally characterized by small sample sizes and short follow-up periods [38,46,49]. Qualitative studies provided additional information on participants’ experiences, perceptions, and contextual factors related to workplace nutrition and occupational well-being [24,47]. Overall, the evidence base was methodologically heterogeneous with respect to study design, reflecting the exploratory nature of the field and limiting direct comparison across studies.

### 3.3. Population and Sample Size

The included studies involved a broad range of healthcare-related populations, including dentists, dental professionals, physicians, nurses, dietitians, healthcare workers, and healthcare students [14,15,24,26,35,38,43,44,45,46,47,48,49]. Most studies included multidisciplinary healthcare populations. Only two studies specifically involved dental professionals, including dental students, practicing dentists, and members of the dental team [26,47]. Sample sizes varied considerably, ranging from 22 participants in qualitative studies to 9196 physicians in a nationwide survey conducted across China [24,35]. The largest quantitative study included physicians from hospitals across 31 provinces, municipalities, and autonomous regions of China [35], whereas qualitative studies and pilot intervention studies generally involved substantially smaller samples [24,38,46,47]. The included studies varied considerably with respect to both population characteristics and sample size.

### 3.4. Data Collection Methods

Data collection methods varied according to study design. Quantitative studies predominantly relied on structured questionnaires, nutrition knowledge assessments, validated survey instruments, and self-reported lifestyle and dietary measures administered through online or self-completed questionnaires [14,15,26,35,43,44,45,46,48,49]. Educational and intervention studies employed pre–post assessment tools to evaluate changes in nutrition knowledge, dietary behaviours, anthropometric measures, and health-related outcomes [38,46,49]. Qualitative studies used focus groups or semi-structured interviews combined with thematic analysis to explore participants’ experiences, perceptions, and workplace-related factors [24,47]. Data collection approaches therefore reflected the diversity of the included study designs.

### 3.5. Emerging Themes Across the Evidence Base

The identified themes were supported by evidence derived from studies with different methodological designs, including cross-sectional, qualitative, educational intervention, pilot intervention, and randomized controlled studies. The identified themes were derived from studies involving diverse healthcare populations, including the two studies conducted among dental professionals. The synthesis of the included studies revealed five recurring themes describing the relationships between workplace dietary behaviours, educational interventions, and occupational well-being among healthcare professionals [14,15,24,26,35,38,43,44,45,46,47,48,49]. Although the methodological approaches varied substantially, consistent patterns emerged across study populations, settings, and outcome measures [14,15,24,26,35,38,43,44,45,46,47,48,49]. These themes informed the proposed conceptual framework describing the relationship between nutrition-related workplace strategies and occupational health outcomes.

#### 3.5.1. Occupational Stress as a Driver of Unhealthy Dietary Behaviors

One of the most consistent patterns identified across the evidence base was the association between occupational stress and unhealthy dietary behaviours among healthcare professionals [14,35,43,44]. Despite differences in study populations, settings, and methodological approaches, the findings consistently indicated that demanding work environments were associated with poorer dietary habits and disrupted eating behaviours [14,15,35]. Workload intensity, prolonged working hours, emotional demands, fatigue, and disrupted work schedules were associated with irregular meal timing, meal skipping, emotional eating, and increased consumption of energy-dense foods [14,15,35,43]. These patterns were particularly evident in studies conducted during or immediately following the COVID-19 pandemic, when healthcare professionals experienced increased occupational demands and psychological strain [14,15,43,45]. Across the included studies, occupational stress frequently co-occurred with unhealthy dietary behaviours, burnout, reduced well-being, and poorer health-related outcomes [14,35,43,44]. Similar findings were also reported among healthcare professionals despite their high level of health-related knowledge, suggesting that workplace, organizational, and behavioural factors may influence dietary practices beyond nutrition knowledge alone [26,45,48].

#### 3.5.2. The Knowledge–Behavior Gap

A commonly reported finding across the included studies was the apparent disconnect between nutrition knowledge and dietary behaviour [24,26,38,47,49]. Although the included studies examined diverse populations—including healthcare students, dentists, physicians, nurses, and other healthcare professionals—the relationship between nutrition knowledge and dietary behaviour showed similar patterns across these groups. However, the specific behavioural, occupational, and environmental factors associated with dietary choices differed according to the study population and setting [15,24,26,38,43,44,45,46,47,48,49].

Educational interventions and greater educational exposure were generally associated with higher levels of nutrition knowledge and awareness [38,49], but these improvements did not consistently translate into healthier food choices or sustained changes in dietary behaviours [26,49]. Among healthcare professionals, including dental students and practicing dentists, attitudes, personal experiences, workplace influences, and lifestyle-related factors were more consistently associated with dietary behaviours than nutrition knowledge alone [24,26,47]. Qualitative findings further highlighted the influence of self-efficacy, role modelling, workplace culture, and social interactions on food-related decisions, emphasizing that healthy dietary behaviours develop within broader behavioural and organizational contexts rather than through nutrition education alone [24,47]. Rather than reflecting nutrition knowledge alone, dietary behaviours were described as being shaped by the interaction of cognitive, behavioural, social, workplace, and environmental factors.

#### 3.5.3. Organizational Environment and Dietary Choices

Organizational characteristics of healthcare workplaces emerged consistently across the included studies as factors associated with dietary behaviours, complementing individual-level determinants such as nutrition knowledge, attitudes, and personal preferences [15,24,26,35,45,46,47,48,49]. Four recurring organizational dimensions were identified across the evidence base, encompassing work organization, workplace food environments, social and organizational culture, and workplace-based interventions.

With regard to work organization, several studies reported associations between workload intensity, shift work, prolonged working hours, work schedules, and eating behaviours [15,35,45]. Physicians working under demanding schedules frequently reported irregular meal timing, shorter meal duration, and greater reliance on meals prepared outside the home, whereas shift workers demonstrated lower adherence to healthier dietary patterns and less regular meal schedules than day workers [15,35]. Occupational characteristics, including patient workload and years of professional experience, were also examined in relation to dietary quality across healthcare settings [45].

A second recurring dimension concerned the workplace food environment. Studies described the availability and accessibility of food, opportunities for meal breaks, eating locations, and workplace food services as components of healthcare settings associated with dietary practices [15,24,35]. Hospital canteens, meals consumed outside the home, limited opportunities for regular meals, and restricted access to healthier food options were reported across different healthcare environments [15,35]. Dietary inadequacies, including insufficient intake of protein, dietary fibre, vitamins, and minerals, were also documented among healthcare professionals despite relatively similar occupational settings [48].

The third dimension was related to social and organizational culture. Qualitative studies described workplace culture, social interactions, peer influence, role modelling, and supportive workplace relationships as elements associated with everyday food-related behaviours [24]. Evidence from the dental studies similarly highlighted communication, collaborative approaches, and workplace action plans as organizational factors associated with healthier workplace practices [47]. Within the dental studies, dietary behaviours differed across dental students, postgraduate trainees, and practicing dentists, while attitudes demonstrated stronger associations with healthy dietary behaviours than nutrition knowledge alone [26,47].

The fourth dimension comprised organizational interventions implemented within healthcare workplaces. Intervention studies incorporated workplace-level components alongside nutrition education, including active-learning educational programmes, individualized nutritional counselling, multidisciplinary collaboration, behavioural support, workplace health-promotion activities, and organizational support delivered within healthcare settings [38,46,49]. Although the structure and duration of these interventions varied, the identified interventions were implemented within organizational settings and combined educational strategies with broader workplace-oriented components.

#### 3.5.4. Multicomponent Workplace Interventions

The intervention studies included in this review described integrated workplace health-promotion approaches that combined nutrition education with complementary behavioural, clinical, and organizational components rather than delivering nutrition education as a stand-alone intervention [38,46,49]. Although the programmes differed in duration, target population, and implementation setting, they shared a multidimensional structure that addressed several aspects of health promotion simultaneously.

Educational activities formed the foundation of all interventions and included structured nutrition education, active-learning methodologies, technology-supported teaching, face-to-face instruction, and individualized dietary counselling [38,49]. These educational components were implemented in both academic and healthcare settings and were designed to strengthen participants’ nutrition knowledge while supporting healthier dietary decision-making [38,49].

Beyond education, interventions incorporated behavioural strategies intended to facilitate the adoption of healthier lifestyle practices. These included individualized counselling, behaviour-change support, dietary guidance, and, in some programmes, the integration of physical activity promotion alongside nutrition interventions [46,49]. Rather than focusing exclusively on knowledge acquisition, the interventions addressed multiple health-related behaviours within the same programme.

Clinical assessment represented another common characteristic of the intervention studies. Participants underwent anthropometric measurements, nutritional assessments, biochemical testing, and evaluation using validated instruments measuring eating behaviours, general health, quality of life, and psychological well-being before and after programme implementation [46,49]. Educational programmes additionally assessed changes in nutrition knowledge using standardized pre- and post-intervention evaluation tools [38].

Finally, implementation was consistently embedded within existing educational or healthcare organizations. Programmes incorporated multidisciplinary collaboration, workplace health-promotion activities, organizational support, and structured delivery within universities or healthcare institutions, including interventions developed according to the Total Worker Health^®^ framework [38,46,49]. Outcome assessment encompassed educational, behavioural, clinical, and psychosocial domains, with studies reporting outcomes related to nutrition knowledge, dietary behaviours, anthropometric indicators, metabolic parameters, general health, and quality of life [38,46,49].

#### 3.5.5. Representation of Dental Professionals in the Evidence Base

Most included studies involved multidisciplinary healthcare populations, whereas only two studies were conducted specifically among dental professionals, including dental students, practicing dentists, oral health therapists, dental assistants, specialists in Special Needs Dentistry, and members of the dental team [14,15,24,35,38,43,44,45,46,48,49]. These studies involved dental undergraduates, postgraduate students, practicing dentists, oral health therapists, dental assistants, specialists in Special Needs Dentistry, and administrative staff working within dental teams [26,47].

The two dental studies addressed a range of topics, including dietary behaviours and their determinants, nutrition knowledge, attitudes towards healthy eating, workplace experiences, and organizational influences affecting professional practice [26,47]. The study conducted in Thailand examined dietary behaviours across different stages of dental education and professional practice, enabling comparisons between undergraduate students, postgraduate students, and practicing dentists [26]. The Australian qualitative study explored perspectives from multiple members of the dental team working in public, private, and mixed practice settings, providing insights into organizational and workplace experiences across different professional roles [47]. In contrast, intervention studies identified in this review were conducted among broader healthcare populations or healthcare students rather than exclusively within dental settings [38,46,49]. Accordingly, only limited evidence was available regarding workplace nutrition interventions specifically targeting dental professionals within the included literature.

## 4. Discussion

### 4.1. Main Findings and Interpretation of the Evidence

The present scoping review suggests that workplace nutrition among healthcare professionals should be understood as a multidimensional component of occupational health rather than solely as individual lifestyle behaviour. Across the included studies, dietary practices were associated with the interplay of occupational demands, behavioural determinants, educational influences, and organizational conditions, suggesting that food choices are influenced by the broader work environment rather than determined exclusively by personal knowledge or motivation. Although the available evidence was methodologically heterogeneous, the convergence of findings across diverse healthcare settings supports an integrated interpretation of workplace nutrition as part of a complex occupational system rather than isolated health behaviour.

One of the most consistent observations emerging from the included studies concerned the influence of occupational stress and work organization on dietary behaviours. Demanding workloads, emotional strain, disrupted schedules, limited opportunities for regular meals, and insufficient recovery time were associated with irregular meal patterns, emotional eating, increased fat intake, poorer dietary quality, and less favourable well-being indicators [14,15,35,43,44,45]. These behaviours should not be interpreted as independent consequences of occupational pressure. Instead, they appear to reflect interconnected behavioural, psychological, temporal, and physiological responses through which demanding working conditions may influence both food choices and health-related outcomes.

An additional dimension emerging from the evidence concerns occupational scheduling and circadian disruption. In a four-year retrospective cohort study, Ye [50] reported an association between shift work and metabolic syndrome, indicating that repeated disruption of working and recovery rhythms may have longer-term metabolic implications. Among healthcare practitioners, Migdanis et al. [51] also identified differences in dietary patterns according to shift-working conditions during the COVID-19 pandemic. Considered together with the findings of the included studies, this evidence suggests that occupational scheduling may influence not only the timing and regularity of eating but also dietary composition and associated health outcomes. Nevertheless, the predominance of cross-sectional evidence within the present review limits causal interpretation, and the relationships identified may also be influenced by sleep, physical activity, occupational role, socioeconomic circumstances, and other lifestyle-related determinants. Although occupational demands differ across healthcare professions, several of the mechanisms identified in the broader healthcare literature may also be relevant to dental practice. Prolonged clinical sessions, tightly scheduled appointments, sustained concentration, continuous patient interaction, and limited opportunities for uninterrupted breaks may similarly influence eating patterns and hydration among dental professionals. However, these observations should be interpreted cautiously because profession-specific longitudinal evidence remains limited.

A second major finding concerned the persistent gap between nutrition knowledge and dietary behaviour. Across the included studies, higher nutrition knowledge or exposure to nutrition education did not consistently correspond to healthier dietary practices [24,26,38,47,49]. This pattern was particularly apparent in studies demonstrating that nutrition knowledge frequently coexisted with workplace barriers, established habits, limited food accessibility, social influences, and unfavourable working routines. The findings therefore suggest that a purely educational model, in which the acquisition of information is assumed to automatically translate into behavioural change, may be insufficient. This interpretation is supported by Khosa et al. [52], who reported that nutrition knowledge was not significantly associated with eating habits among healthcare workers, despite moderate or high knowledge levels among most participants. Similarly, Ferreira-Pêgo et al. [53] found that eating behaviour reflected the combined influence of nutrition knowledge, age, and adherence to a Mediterranean dietary pattern. At a broader level, the systematic review by Spronk et al. [54] identified a positive but generally modest relationship between nutrition knowledge and dietary intake, while also documenting substantial variation in how both constructs were measured. These observations suggest that nutrition knowledge constitutes only one element of dietary behaviour, the adoption and maintenance of which appear to depend on a broader interplay of behavioural, occupational, social, and organizational influences.

The available evidence suggests that dietary practices are influenced by multiple interacting determinants, including personal attitudes, habitual behaviours, occupational demands, social influences, environmental opportunities, and organizational support. These findings have important implications for the design of workplace interventions: educational programmes may improve awareness and decision-making capacity, but their behavioural impact depends on whether professionals are able to apply this knowledge within the temporal, social, and material conditions of their workplace. Hyży et al. [55] addressed this issue through a participatory workplace intervention focused not only on nutrition information but also on perceived dietary barriers and healthy-eating intentions. Although its pilot design and short duration preclude conclusions regarding sustained effectiveness, the study illustrates how educational strategies can be linked to practical barriers, employee participation, and the contexts in which food-related decisions occur.

The thematic synthesis also supports a conceptual shift from viewing workplace nutrition primarily as an issue of individual responsibility towards understanding it as a shared individual and organizational concern. Across the included studies, dietary practices were associated with scheduling, meal regularity, food accessibility, eating location, workplace relationships, professional culture, and organizational support [15,24,26,35,44,45,46,47,48,49]. The included evidence suggests that opportunities for healthy eating within healthcare workplaces are influenced by organizational factors, including meal timing, food accessibility, workload distribution, and protected breaks. Consequently, healthy dietary choices may depend not only on individual motivation but also on whether the working environment enables these choices to be translated into routine practice. This interpretation is consistent with the systematic review by Clohessy et al. [56], which identified interacting individual, social, physical-environmental, and organizational influences on employees’ eating behaviours. Importantly, workplace food choices were not explained by a single determinant but reflected the combined effects of workload, convenience, food availability, social norms, occupational routines, and organizational practices. This broader evidence supports the interpretation that workplace dietary behaviour is context-dependent and that intervention strategies should target the conditions surrounding food choices as well as individual motivation. Policy and implementation research further strengthens this systems-oriented interpretation. Hyseni et al. [57] demonstrated that dietary patterns may be influenced through structural policy actions addressing food availability, affordability, composition, and promotion. Although their review primarily concerned population-level policies, its findings support the broader principle that dietary behaviour is responsive to environmental and structural conditions. At the workplace level, Rosin et al. [58] identified tools and resources used to implement healthy food and drink policies, including implementation guidance, environmental assessment, communication materials, monitoring processes, and organizational support. Their synthesis indicates that the formal adoption of a policy is unlikely to modify behaviour unless it is accompanied by practical implementation capacity and sustained institutional commitment.

The intervention evidence similarly suggests that workplace nutrition programmes should be interpreted as heterogeneous organizational strategies rather than as a uniform category of intervention. The meta-analysis and meta-regression conducted by Melián-Fleitas et al. reported favourable associations between workplace nutrition-, food-, and diet-related interventions and selected dietary and health outcomes, while also demonstrating considerable variation across intervention characteristics and study contexts [59]. This heterogeneity is important because programme effects are likely to depend on intervention intensity, duration, participant engagement, the combination of components, and the extent to which the programme is integrated into everyday organizational practice. Evidence specifically involving healthcare workers provides additional support for this interpretation. The systematic review and meta-analysis by Panchbhaya et al. [37] found that workplace dietary interventions could improve selected aspects of healthcare workers’ dietary intake, while also highlighting substantial variation in intervention design, implementation, and measured outcomes. The relevance of this review to the present synthesis lies in its healthcare-specific population and its indication that modifying dietary intake among healthcare personnel requires more than the passive provision of nutrition information.

Across these reviews, a recurring pattern is that workplace interventions commonly combine educational components with behavioural and organizational strategies rather than delivering education as a stand-alone approach. This interpretation is reflected in the intervention studies included in the present review. The identified programmes incorporated nutrition education together with individualized counselling, behavioural support, psychological assessment, physical activity promotion, clinical monitoring, or organizational health-promotion components [38,46,49]. The evidence does not establish the superiority of multicomponent programmes over stand-alone education, because direct comparative studies were scarce. It does, however, show that the available interventions generally addressed several determinants of dietary and health behaviour simultaneously and assessed outcomes extending beyond knowledge acquisition to behavioural, clinical, psychosocial, and organizational domains. This multilevel interpretation is further supported by the systematic review of Grimani et al. [60], which examined workplace nutrition and physical activity interventions incorporating changes to the physical work environment or organizational structure. The review reported that some interventions were associated with improvements in work-related outcomes, particularly absenteeism, although the authors emphasized the need for higher-quality controlled studies, objective outcome measures, and longer follow-up. These findings broaden the discussion beyond dietary behaviour by indicating that organizationally embedded health-promotion programmes may also be associated with outcomes relevant to workforce sustainability and professional performance. Similarly, the systematic review by Melnyk et al. [3] identified diverse interventions targeting mental health, physical health, well-being, and lifestyle behaviours among physicians and nurses. Variation in intervention components and reported outcomes indicates that no single educational or behavioural strategy can be expected to address all dimensions of professional health. The available evidence suggests that workplace interventions may benefit from being tailored to the determinants operating within specific workplace contexts, integrating individual-level support with environmental and organizational strategies where appropriate.

**Figure 2:**
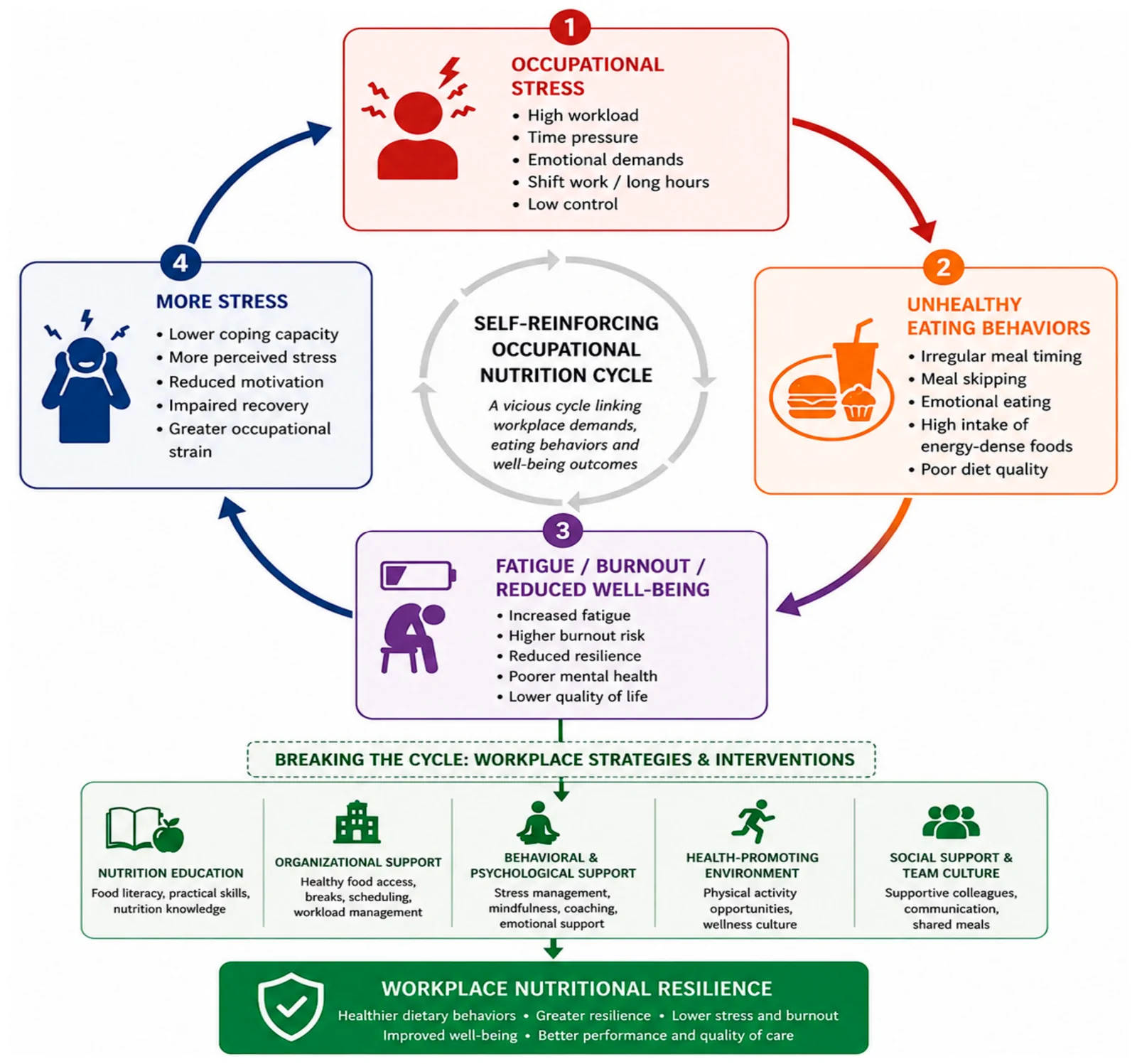
Proposed conceptual framework of Workplace Nutritional Resilience. The framework integrates the principal themes identified in this scoping review and illustrates the potential interactions among occupational stress, workplace conditions, dietary behaviours, and organizational support. It is intended as a conceptual interpretation of the available evidence to inform future research and workplace nutrition interventions rather than as an empirically validated model.

These observations provide the theoretical foundation for the conceptual framework of Workplace Nutritional Resilience proposed in the present review (Figure 2). The proposed framework should therefore be interpreted as an evidence-informed conceptual synthesis rather than a directly validated empirical model. In this context, nutritional resilience is conceptualised not simply as an individual’s ability to maintain healthy dietary behaviours despite occupational pressures, but as an emergent property of healthcare systems that support healthy choices through educational, behavioural, and organizational mechanisms. This interpretation extends existing workplace health-promotion models by positioning nutrition as a central component of occupational resilience and provides a conceptual basis for future intervention research in dentistry and other healthcare professions.

### 4.2. Implications for Dental Practice

Although the evidence synthesized in the present review primarily originates from broader healthcare populations, its findings may have important implications for contemporary dental practice. Dentistry is characterized by sustained cognitive demands, prolonged clinical procedures, repetitive fine-motor tasks, continuous patient interaction, and tightly scheduled appointments, creating a work environment in which professionals frequently prioritize patient care over their own physiological needs. Occupational burnout and work-related stress are well-recognized challenges within the dental profession [19,20,61], and these conditions provide a plausible context in which regular meals, adequate hydration, and other self-care behaviours may be compromised during routine clinical practice.

From this perspective, unhealthy dietary behaviours should not be interpreted simply as individual lifestyle choices or personal failures to adopt healthier habits. Rather, they may reflect adaptive responses to demanding clinical environments in which operational pressures, time constraints, and organizational routines influence everyday opportunities for eating. This interpretation is consistent with the findings of the present review, which indicate that nutrition knowledge alone does not consistently translate into healthier dietary practices [26,52,54]. Instead, dietary behaviour appears to emerge from the interaction between individual motivation and the organizational context in which clinical work is delivered. Within dental practice, appointment scheduling, workload intensity, opportunities for uninterrupted breaks, food accessibility, practice culture, and leadership support may collectively determine whether healthy dietary intentions can realistically be maintained throughout the working day [56,58].

Although dentists are generally less exposed to rotating shift work than hospital-based healthcare professionals, the organizational mechanisms identified across the broader healthcare literature may also be applicable to dental practice. Consecutive patient appointments, prolonged chairside activity, sustained concentration, missed meal opportunities, and insufficient recovery periods may create patterns of occupational strain comparable to those reported in other healthcare settings [50,51]. Nevertheless, caution should be exercised when extrapolating evidence directly to dentistry, as two of the three dental studies included in the present review involved both dental students and practicing professionals. Because students are primarily exposed to academic demands, whereas practicing dentists must simultaneously manage clinical responsibilities, patient expectations, practice organization, and business-related pressures, the determinants of dietary behaviours and occupational well-being may differ substantially across these populations. This highlights the need for future research focusing specifically on practicing dental professionals.

The concept of Workplace Nutritional Resilience proposed in the present review provides a framework for interpreting these relationships from a systems perspective. Rather than conceptualising nutritional resilience solely as an individual’s capacity to maintain healthy dietary behaviours despite occupational pressures, the framework emphasizes the ability of the workplace itself to create conditions that facilitate healthy choices. In this context, nutritional resilience emerges through the interaction of individual behaviours, organizational support, workplace culture, and operational structures that either enable or constrain healthy eating during everyday clinical practice. This interpretation is consistent with contemporary workplace health promotion models, which increasingly recognize that sustainable behavioural change is more likely when educational, behavioural, and organizational strategies are implemented simultaneously [3,37,60].

These observations have important practical implications for dental practice. Improving dietary behaviours is unlikely to depend on nutrition education alone, despite its essential role in strengthening knowledge and awareness. Sustainable behavioural change will probably require organizational measures that make healthier choices both accessible and feasible within the realities of clinical practice. Protected meal breaks, hydration-supportive workplaces, realistic appointment scheduling, leadership practices that encourage self-care, and team-based well-being initiatives may all contribute to strengthening workplace nutritional resilience [37,55,56,62]. In parallel, closer collaboration between dental professionals and dietitians may further strengthen both preventive patient care and nutrition awareness within the dental team through integrated and interdisciplinary models of practice [25].

Finally, the proposed conceptual framework should be regarded as an evidence-informed model requiring prospective validation in dentist-specific populations. Future longitudinal and intervention studies are needed to investigate whether organizational strategies designed to support healthy dietary behaviours can improve not only nutrition-related outcomes but also occupational well-being, resilience, workforce sustainability, and ultimately the quality of patient care delivered within modern dental practice.

### 4.3. Strengths and Limitations

This scoping review provides an updated synthesis of the available evidence regarding workplace nutrition, dietary behaviours, and occupational well-being among healthcare professionals. A major strength of the review is the integration of nutritional, behavioural, psychological, educational, and organizational perspectives, allowing workplace nutrition to be examined within the broader context of occupational health and healthcare system performance rather than as an isolated lifestyle behaviour. Furthermore, the review proposes the concept of Workplace Nutritional Resilience as an evidence-informed conceptual framework that integrates the principal themes identified across the included studies and offers a foundation for future hypothesis generation and intervention design. Nevertheless, several limitations should be acknowledged. First, the included studies were methodologically heterogeneous with respect to study design, study populations, interventions, and outcome measures, precluding quantitative synthesis and limiting direct comparisons across studies. Second, most studies employed cross-sectional designs and relied on self-reported data, limiting causal inference and increasing the potential for recall, reporting, and social desirability bias. Third, relatively few studies focused specifically on dental professionals, reducing the direct applicability of some findings to contemporary dental practice. In addition, two of the three studies involving dental populations included dental students alongside practicing professionals. Given the substantial differences between educational and clinical workplace environments, the determinants of dietary behaviours and occupational well-being may not be directly comparable across these populations, limiting the generalizability of the available dental-specific evidence. An additional limitation is that studies were selected according to their publication date rather than the timing of data collection. Consequently, some studies published during the selected review period may have included data collected before or during the COVID-19 pandemic. This should be considered when interpreting the findings, although the review was designed to synthesize contemporary evidence rather than evaluate pandemic-specific effects. Finally, only peer-reviewed publications written in English were included. Grey literature was not searched because the review aimed to synthesize evidence from peer-reviewed scientific publications using predefined eligibility criteria. Nevertheless, excluding grey literature may have resulted in the omission of relevant workplace programmes or educational initiatives not reported in indexed journals and should be considered a limitation of the present review. These limitations should also be considered when interpreting the proposed Workplace Nutritional Resilience framework, which should be regarded as an evidence-informed conceptual synthesis rather than an empirically validated model. Despite these limitations, the review identifies common patterns across the available literature and provides a conceptual foundation for future research, educational initiatives, and workplace health promotion strategies in dentistry and the wider healthcare sector.

### 4.4. Future Research Directions

Future research should prioritize longitudinal and intervention studies across healthcare professions, with particular emphasis on practicing dental professionals, to better understand the relationships among dietary behaviours, occupational stress, burnout, resilience, and professional well-being. Standardized measures of dietary quality, resilience, burnout, and occupational outcomes would improve comparability across studies and strengthen the evidence base. Future studies should also evaluate multicomponent workplace interventions integrating nutrition education with organizational, behavioural, and psychological strategies, while examining how workplace culture, scheduling practices, food accessibility, leadership support, and protected recovery opportunities influence dietary behaviours in dental settings. Such evidence will be essential to validate the proposed Workplace Nutritional Resilience framework and to support the development of sustainable, evidence-based interventions that enhance the health, well-being, and long-term sustainability of the dental workforce.

## 5. Conclusions

This scoping review synthesizes the currently available evidence regarding workplace nutrition, dietary behaviours, and occupational well-being among dentists and healthcare professionals. Although the available evidence is preliminary and methodologically heterogeneous, it suggests that dietary behaviours in healthcare settings are shaped by a complex interplay of individual, behavioural, occupational, and organizational factors rather than nutrition knowledge alone. Workplace nutrition should therefore be considered an integral component of occupational health and workforce sustainability rather than solely an individual lifestyle behaviour. Based on the thematic synthesis, we propose the concept of Workplace Nutritional Resilience as an evidence-informed conceptual framework that integrates the principal themes identified across the included studies. Although this framework should not be interpreted as an empirically validated model, it provides a conceptual foundation for future research and the development of multicomponent workplace interventions that combine nutrition education with behavioural and organizational strategies. The current evidence remains limited by methodological heterogeneity, the predominance of observational studies, and the relatively small number of studies specifically involving dental professionals. Future longitudinal and intervention studies across healthcare professions, particularly among practicing dental professionals, are needed to validate the proposed framework and determine whether organizational approaches to workplace nutrition can improve professional well-being, workforce sustainability, and ultimately the quality of patient care.

## Figures and Tables

**Table 1:** Characteristics of studies included in the study.

Authors (Year)	Type of Study/Setting	Population (N)	Exposure/Intervention	Comparators	Limitations	Confounders/Adjustments	Key Outcomes
1. Yaman & Hocaoğlu., 2023 [14]	Cross-sectional online survey conducted in public hospitals (Turkey) (1) *	405 healthcare professionals (69.9% female; specialists, dentists, general practitioners, residents, and other hospital staff; mean age 39 years)	Perceived Stress Scale (PSS), Emotional Eating Scale (EES), Nutrition Change Process Scale (NCPS), and self-reported dietary and nutritional behaviours	Participants reporting changes in eating habits versus those reporting no dietary changes	Cross-sectional design; self-reported data; snowball sampling; online questionnaire; conducted during the COVID-19 pandemic; causal relationships cannot be inferred	Independent *t*-tests, χ^2^ tests, Pearson correlation, univariate and multivariate logistic regression	Overall, 58% of healthcare professionals reported changes in eating and nutritional habits, while 51.4% experienced weight gain during the pandemic. Higher perceived stress (PSS: 27.9 vs. 24.4; *p* < 0.001), emotional eating (EES: 11.89 vs. 8.99; *p* < 0.001), and nutrition change process scores (NCPS: 109.95 vs. 95.37; *p* < 0.001) were observed among participants reporting dietary changes. Economic concerns (OR = 2.55; 95% CI: 1.69–3.84; *p* < 0.001) and concerns about food and water availability (OR = 2.10; 95% CI: 1.39–3.18; *p* < 0.001) were associated with dietary changes in univariate analyses, while losing a relative to COVID-19 emerged as the strongest independent predictor of altered eating behaviours (adjusted OR = 29.5; 95% CI: 2.23–389; *p* = 0.010).
2. Wolska et al. (2022) [15]	Cross-sectional study conducted in West Pomeranian, Poland (1) *	445 healthcare workers (193 shift workers; 252 day workers; physicians, nurses, and paramedics)	Dietary patterns assessed using the validated FFQ-6^®^ questionnaire; Principal Component Analysis (PCA) identified three dietary patterns; Polish-adapted Mediterranean Diet (Polish-aMED^®^) score and percentage of energy from dietary fat (Pfat) were calculated	Shift workers compared with day workers	Cross-sectional design; self-reported dietary intake; online data collection during the COVID-19 pandemic; participants recruited from one Polish region; causality cannot be established	Principal Component Analysis (PCA); logistic regression (crude and adjusted models); χ^2^ tests; Kruskal–Wallis tests	Compared with day workers, shift workers had significantly greater adherence to the “Meat–fats–alcohol–fish” dietary pattern (adjusted OR = 2.38; 95% CI: 1.27–4.47; *p* < 0.01), higher odds of consuming >35% of total energy from fat (OR = 1.73; 95% CI: 1.06–2.83; *p* < 0.05), and lower adherence to a Mediterranean dietary pattern (Polish-aMED^®^ score: OR = 0.57; 95% CI: 0.33–0.98; *p* < 0.05). Shift workers were also less likely to report regular mealtimes (OR = 0.54; 95% CI: 0.33–0.89; *p* < 0.05) and showed lower adherence to the “Lacto-ovo-vegetarian” dietary pattern (OR = 0.48; 95% CI: 0.26–0.89; *p* < 0.05).
3. Chen et al., 2023 [35]	Cross-sectional nationwide survey conducted in hospitals across mainland (China) (1) *	9196 physicians from hospitals in 31 provinces, municipalities and autonomous regions of China	Self-administered questionnaire assessing eating habits (meal source, frequency of home-cooked meals, meal regularity, eating duration), perceived suboptimal health status (SHS), BMI classification, micronutrient deficiency-related diseases, obesity and metabolic diseases	Comparisons according to eating habits (eating out vs. home meals, regular vs. irregular meals, meal duration) and physician specialty	Cross-sectional design; self-reported questionnaire; predominance of female participants (83.3%); self-reported BMI and health conditions; causality cannot be established	Pearson χ^2^ tests and multivariable logistic regression adjusted for age, sex, working hours, sleep quality, physical activity, sedentary time, smoking and alcohol consumption	Among physicians, 53.4% primarily ate in hospital canteens, 23.0% ate in restaurants/takeaways, 30.5% reported irregular meals, and 34.6% consumed meals in <10 min. Overall, 56.6% reported suboptimal health status, 31.5% were overweight/obese, and dyslipidaemia was reported by 21.5%. Compared with physicians eating at home, dining out was associated with higher odds of suboptimal health (adjusted OR = 1.75; 95% CI: 1.52–2.01; *p* < 0.001), while irregular meals were associated with more than twofold higher odds (adjusted OR = 2.44; 95% CI: 2.19–2.72; *p* < 0.001). Eating meals in <10 min was also independently associated with suboptimal health (adjusted OR = 2.25; 95% CI: 1.67–3.04; *p* < 0.001).
4. Mancin et al., 2023 [38]	Pilot pre–post observational study conducted at a university in Northern Italy (Italy) (2) *	28 Master of Science in Nursing and Midwifery (MScN) students (mean age 34.0 ± 6.4 years; 89.3% female; 17.9% had previously attended a nutrition course)	Clinical nutrition educational module based on active learning methodologies (critical thinking, ICT-supported teaching, and face-to-face learning); nutritional knowledge assessed using a validated Italian nutrition questionnaire before and after the intervention	Pre-intervention versus post-intervention knowledge scores	Small sample size; single-centre study; absence of a control group; limited generalisability; pilot design	Multilevel mixed-effects regression adjusted for age, sex, degree grade and previous nutrition education; subgroup analyses of basic and clinical nutrition knowledge	Participation in the active-learning module significantly improved nutritional knowledge, with the mean questionnaire score improving from 20.25 ± 2.68 before training to 16.75 ± 1.85 after training (mean difference −3.50; 95% CI −4.59 to −2.40; *p* < 0.001). Correct responses for basic nutrition increased from 59.6% to 83.9% (*p* < 0.001), while clinical nutrition knowledge improved from 75.7% to 97.1% (*p* < 0.001). Demographic characteristics and previous nutrition education were not significant confounders of learning outcomes.
5. Gilbert et al., 2023 [43]	Cross-sectional survey conducted at a large Midwestern university and affiliated healthcare system in the United States (USA) (1) *	University faculty, staff, and healthcare workers	Self-reported assessment of physical activity, dietary behaviours, and well-being using an online survey	Comparisons according to physical activity, dietary behaviours, occupational role, and work setting	Cross-sectional design; self-reported data; convenience sampling; potential selection and recall bias; causal relationships cannot be established	Descriptive statistics, χ^2^ tests, *t*-tests, and multivariable regression analyses	Lower physical activity and poorer dietary behaviours were associated with poorer well-being, whereas participants maintaining healthier lifestyle behaviours reported better physical and psychological well-being, supporting workplace health promotion initiatives during the COVID-19 pandemic.
6. Vasquez-Purí et al. (2023) [44]	Cross-sectional study conducted in a public hospital, Rioja, San Martín, Peru (2021) (1) *	300 healthcare professionals (physicians, nurses, nutritionists/dietitians, and technical staff)	Burnout assessed using the validated Maslach Burnout Inventory (22 items); habitual fat intake assessed using the validated Block Fat Screener; BMI classified according to WHO criteria	Comparisons by gender and multivariable analyses adjusted for age, gender, origin, diet, and profession	Cross-sectional design; convenience sampling; single hospital; self-reported dietary intake; inability to infer causality	Pearson correlation coefficients; multivariable linear regression (crude and adjusted models); χ^2^/Fisher’s exact tests; Wilcoxon rank-sum tests	Women reported significantly higher burnout (27.5% vs. 15.8%, *p* = 0.014) and high fat intake (25.9% vs. 13.9%, *p* = 0.039) than men. Burnout was positively associated with fat intake after adjustment (β = 1.10; 95% CI: 0.57–1.62; *p* < 0.001). Similar associations were observed in women (β = 1.17; 95% CI: 0.49–1.85; *p* < 0.001) and men (β = 1.08; 95% CI: 0.16–2.00; *p* = 0.022). Among participants with high fat intake scores (>20), burnout increased linearly with fat consumption. No statistically significant association was found between burnout and BMI (*p* > 0.05).
7. Hobby et al., 2024 [24]	Cross-sectional qualitative study using semi-structured interviews (telephone/videoconference) (Australia) (3) *	22 healthcare professionals with direct patient contact, including dietitians and other allied health professionals	Exploration of healthcare professionals’ perceptions of how their personal dietary behaviours influence self-efficacy in providing nutrition care	Comparison of perceptions across different professional backgrounds	Qualitative design; small purposive sample; self-reported perceptions; conducted in one country; findings may not be generalisable	Thematic analysis of semi-structured interview transcripts	Three main themes emerged: (1) personal life experiences shaped confidence (self-efficacy) in delivering nutrition care; (2) continuous observation and role modelling influenced nutrition-related attitudes and professional practice; and (3) workplace social interactions both facilitated and hindered healthy eating behaviours through social support and social pressure. Participants perceived that improving their own dietary behaviours could enhance the quality of nutrition care they provide, highlighting the importance of supportive workplace food environments.
8. Tantimahanon et al. (2024) [26]	Cross-sectional questionnaire-based observational study (Thailand) (1) *	Dental undergraduates (n = 264), postgraduate students (n = 247), and practicing dentists (n = 331); total sample n = 842. Mixed/not specified (nationwide sample of licensed dentists).	Assessment of dietary behaviours and determinants influencing eating habits (knowledge, attitudes, dietary behaviours, lifestyle factors)	Comparisons across educational and professional groups (UG vs. PG vs. DT)	Cross-sectional design; self-reported questionnaire; potential recall, social desirability and selection bias	Regression models adjusted for demographic and lifestyle variables (age, sex, alcohol consumption, exercise, stress, living arrangements, family dietary habits, etc.)	Attitude was the strongest predictor of healthy dietary behaviours across all groups (UG β = 0.370; PG β = 0.512; DT β = 0.642; all *p* < 0.001). Alcohol consumption was negatively associated with healthy dietary behaviours (UG β = −0.135; PG β = −0.220; DT β = −0.216; all *p* < 0.001). Knowledge showed only a weak positive association, significant only among postgraduate students (β = 0.143, *p* = 0.006). Significant differences in dietary behaviours were observed across educational levels, supporting the need for tailored interventions.
9. Znyk & Kaleta, (2024) [45]	Cross-sectional study conducted in primary healthcare settings, Poland (2020–2022) (1) *	492 healthcare professionals: 161 General Practitioners (GPs) and 331 nurses	Assessment of dietary quality using the validated Dietary Quality Score (DQS) together with demographic, occupational and lifestyle characteristics	Comparison between GPs and nurses and according to demographic and occupational variables	Cross-sectional design; self-reported dietary habits, weight and height; conducted during the COVID-19 period; single geographic region; no income data; potential recall bias	Univariable and multivariable logistic regression adjusting for demographic, occupational and lifestyle variables	Unhealthy dietary habits were identified in 25.8% of participants (17.4% of GPs vs. 29.9% of nurses), while only 12.2% demonstrated healthy dietary habits (19.2% of GPs; 8.8% of nurses). More than 57% consumed less than the recommended amount of fish, 70.7% used unhealthy fats for spreading, and only 27.2% consumed >5 vegetable servings/week. Univariable analyses showed that smoking (OR = 1.58), seeing ≤100 patients/week (OR = 1.59), and longer work experience among nurses (OR = 1.81 for >20 years) were associated with higher odds of unhealthy dietary habits, although these associations were not retained in multivariable analyses. Lower fish intake and unhealthy cooking fats were also associated with greater odds of overweight/obesity.
10. Di Prinzio et al., 2025 [46]	Prospective pilot pre–post study conducted at a tertiary pediatric hospital in Rome (Italy) (4) *	51 healthcare workers (78.4% female; mean age 52.0 ± 8.9 years; mainly nurses [56.9%]; 54.9% obese and 43.1% overweight)	Food Education Program (FEP) based on the Total Worker Health^®^ approach, combining individualized nutritional counselling, psychological support, anthropometric and biochemical assessments, and validated questionnaires (PIRI, GHQ-12, SF-36, EAT-26)	Pre-intervention versus post-intervention outcomes; comparisons between obese and overweight participants; gender subgroup analyses	Pilot study; single-centre design; voluntary recruitment with potential selection bias; relatively small sample; no randomized control group	Non-parametric pre–post analyses (SPSS); Mann–Whitney U tests for subgroup comparisons; Return on Investment (ROI) analysis based on sickness absence days	The intervention achieved a 32.1% success rate, with significant improvements in waist-to-hip ratio (*p* < 0.001), glycated haemoglobin (*p* = 0.047), total cholesterol (*p* < 0.001), LDL cholesterol (*p* = 0.001), and vitamin D levels (*p* = 0.002). General health (GHQ-12, *p* < 0.001) and quality of life (SF-36, *p* = 0.011) also improved significantly. Participants experienced a mean reduction of 3.7 sickness absence days during the following year, corresponding to a Return on Investment (ROI) of 6.97, indicating both clinical and organizational benefits of the programme.
11. Malik et al. (2025) [47]	Qualitative study using focus groups and semi-structured interviews (Australia) (3) *	34 dental team members: general dentists (n = 10), oral health therapists (n = 5), Special Needs Dentistry (SND) specialists (n = 8), dental assistants (n = 9), and receptionists (n = 2). Participants were employed in public (67.6%), private (8.8%), or mixed public/private (23.5%) dental settings.	Exploration of dental professionals’ and support staff perspectives and experiences regarding weight stigma in dental settings and identification of strategies to reduce stigma	Comparison of perspectives across different professional roles (general dental team vs. SND specialists vs. support staff)	Qualitative design; relatively small sample; predominantly female participants; participants mainly from one Australian region (except SND specialists); findings may not be generalisable	Thematic (inductive) qualitative analysis; no statistical adjustment	Three major themes emerged: (1) observed experiences of verbal and non-verbal weight stigma in dental settings; (2) perceived impact of weight stigma on dental management, particularly preventive discussions and patient communication; and (3) recommended stigma-reduction strategies centred on team-based education, communication training, advocacy by SND specialists, and development of workplace action plans to improve patient-centred care.
12. Plyassovskaya et al. (2025) [48]	Descriptive cross-sectional study conducted in six primary healthcare clinics, Kazakhstan (2023–2024) (1) *	202 healthcare professionals: 22 male physicians, 67 female physicians, and 113 female nurses	Anthropometric assessment (Tanita BC-418MA bioelectrical impedance) and repeated 24 h dietary recalls evaluating macronutrient and micronutrient intake	Comparisons between male physicians, female physicians, and female nurses	Cross-sectional design; single geographic region; relatively small sample of male physicians; self-reported dietary recalls; no causal inference	Kruskal–Wallis test with Dunn’s post hoc comparisons; Wilcoxon signed-rank tests for comparison with dietary recommendations	Despite predominantly normal BMI values (median BMI ≈ 24.3–24.7 kg/m^2^), participants demonstrated widespread nutritional inadequacies. Protein intake was below recommendations in all groups (53.5–66.5 g/day), carbohydrate intake was significantly below recommended levels (199.5–268.8 g/day), and dietary fibre intake was markedly insufficient (11.3–13.8 g/day). Calcium (376–436 mg/day), potassium (1599–1786 mg/day), magnesium (181–194 mg/day), phosphorus (801–922 mg/day), and vitamins B_1_, B_2_, PP, and C were also consistently below recommended intakes, whereas sodium intake was excessive (2609–3131 mg/day). Iron intake was significantly deficient among women but exceeded recommendations in male physicians. No statistically significant differences in overall dietary patterns were observed between physicians and nurses, suggesting that organizational rather than professional factors may predominantly influence dietary behaviours
13. Colaprico et al. (2026) [49]	Four-arm multicentre randomized controlled trial (Italy) (5) *	Overweight/obese healthcare workers and university employees; 87 randomized; 61 completed the study (63.2% women; mean age 51.2 years)	Individualized Mediterranean low-calorie diet alone or combined with educational nutrition videos, physical activity materials, or both over a 3-month intervention	Four parallel groups: A: diet only; B: diet + nutrition videos; C: diet + physical activity programme; D: diet + nutrition + physical activity	Preliminary analysis; relatively small sample; 29.9% dropout; short follow-up (3 months); self-reported behavioural outcomes; no objective physical activity assessment	Randomization stratified by sex and workplace; baseline groups were comparable in age, BMI, profession and occupational characteristics	Mean body weight decreased by 3.55 kg from baseline to 3 months, with the greatest reduction in Group D (−4.01 ± 4.87 kg). BMI decreased significantly over time. Waist circumference decreased by 5.85 ± 5.07 cm. Healthy dietary behaviours improved, including reduced fast-food, processed meat, sweets and red meat consumption, and increased fruit, legumes and whole-grain intake. Knowledge of the Mediterranean diet improved mainly in Group B (*p* = 0.053, borderline significance). No significant improvement was observed in overall Mediterranean diet adherence (*p* = 0.454) or total physical activity levels.

* Study design: (1) Cross-sectional study; (2) educational intervention study; (3) qualitative study; (4) pilot pre–post intervention study; (5) randomized controlled trial. Abbreviations: β: standardized regression coefficient; BMI: Body Mass Index; CI: Confidence Interval; COVID-19: Coronavirus Disease 2019; DQS: Dietary Quality Score; DT: Dentists; EAT-26: Eating Attitudes Test-26; EES: Emotional Eating Scale; FEP: Food Education Program; FFQ-6^®^: Food Frequency Questionnaire-6^®^; GHQ-12: 12-item General Health Questionnaire; GP: General Practitioner; ICT: Information and Communication Technology; LDL: Low-Density Lipoprotein; MScN: Master of Science in Nursing and Midwifery; NCPS: Nutrition Change Process Scale; OR: Odds Ratio; PCA: Principal Component Analysis; PG: Postgraduate Students; PIRI: Psychophysical Inventory of Resistance and Intolerance; PSS: Perceived Stress Scale; ROI: Return on Investment; SF-36: 36-item Short Form Health Survey; SHS: Suboptimal Health Status; SND: Special Needs Dentistry; SPSS: Statistical Package for the Social Sciences; UG: Undergraduate Students; WHO: World Health Organization; χ^2^: chi-square test; *p*: probability value.

## Data Availability

No new primary data were generated in this study. All data analyzed and synthesized in this scoping review were derived from the published studies included in the review and are summarized in the manuscript and its Supplementary Materials. Data sharing is not applicable to this article.

## Supplementary Materials

The following supporting information can be downloaded at https://www.mdpi.com/article/10.3390/healthcare14172699/s1, Table S1: PRISMA-ScR Checklist.
