# Supplementary material for: Educational Workplace Dietary Interventions and Occupational Well-Being Among Healthcare Professionals: A Scoping Review
Source: Healthcare (Basel). 2026 Aug 24;14(17):2699. doi: 10.3390/healthcare14172699 (PMC13565247; doi:10.3390/healthcare14172699)
Supplement: Supplementary file 1 [file healthcare-14-02699-s001.zip › healthcare-4413441-supplementary.pdf]

Table S1. PRISMA-ScR Checklist

Section	Item	PRISMA-ScR Checklist Item	Reported on page(s)
TITLE	1	Identify the report as a scoping review.	Title page
ABSTRACT	2	Provide a structured summary including background, objectives, eligibility criteria, information sources, charting methods, results, and conclusions.	Abstract
INTRODUCTION	3	Describe the rationale for the review in the context of existing knowledge and explain why the review questions are suited to a scoping review approach.	Introduction
	4	State the objectives and/or questions addressed by the review with reference to key elements such as population, concept, and context.	End of Introduction
METHODS	5	Indicate whether a review protocol exists and, if available, provide registration information. If no protocol was developed, this should be stated.	Not applicable (no protocol registered)
	6	Specify eligibility criteria for sources of evidence, including characteristics such as years considered, language, publication status, and rationale.	Section 2.3
	7	Describe all information sources used in the search and the date the search was last executed.	Sections 2.1–2.2
	8	Present the complete electronic search strategy for at least one database, including any limits used, so that it could be repeated.	Section 2.2; Supplementary Material
	9	Describe the process for selecting sources of evidence, including screening and eligibility assessment.	Section 2.4
	10	Describe the data charting process, including whether charting was performed independently or in duplicate and any calibration procedures.	Section 2.4
	11	List and define all variables for which data were sought and describe any assumptions or simplifications made.	Sections 2.4–2.5
	12	If critical appraisal of individual sources of evidence was undertaken, provide the rationale, methods used, and how this information was used. If not undertaken, state this explicitly.	Section 2.5
	13	Describe the methods used for handling and summarizing the charted data.	Section 2.5
RESULTS	14	Report the numbers of sources screened, assessed for eligibility, included in the review, and reasons for exclusion, ideally using a flow diagram.	Section 2.6; Figure 1
	15	Present characteristics of the included sources of evidence and provide citations.	Table 1; Section 3.1
	16	If critical appraisal was undertaken, present the results.	Not applicable
	17	Present the relevant data charted from each included source of evidence.	Table 1
	18	Summarize and present the charting results in relation to the review objectives and questions.	Sections 3.1–3.5
DISCUSSION	19	Summarize the main results, including an overview of concepts, themes, and types of evidence available, and consider their relevance to the review questions and	Sections 4.1–4.5

		objectives.	
	**20**	Discuss the limitations of the scoping review process.	Section 4.4
**CONCLUSIONS**	**21**	Provide a general interpretation of the results with respect to the review questions and objectives, together with potential implications and/or next steps.	Section 5
**FUNDING**	**22**	Describe sources of funding for the included sources of evidence, where applicable, and describe funding for the scoping review.	Funding statement
